# Supplementary material for: Structured Illumination Microscopy Improves Spot Detection Performance in Spatial Transcriptomics
Source: Cells. 2023 May 4;12(9):1310. doi: 10.3390/cells12091310 (PMC10177490; doi:10.3390/cells12091310)
Supplement: Supplementary file 1 [file cells-12-01310-s001.zip › cells-2282975-supplementary.pdf]

SUPPLEMENTARY MATERIAL

Structured illumination microscopy improves spot detection performance in spatial transcriptomics

Alejandro Linares [1], Carlo Brighi [2], Sergio Espinola [1], Francesco Bacchi [2] and Álvaro H. Crevenna [1,*]

[1] Epigenetics and Neurobiology Unit, European Molecular Biology Laboratory, Rome, Italy
[2] CrestOptics, Rome, Italy
* Correspondence: alvaro.crevenna@embl.it

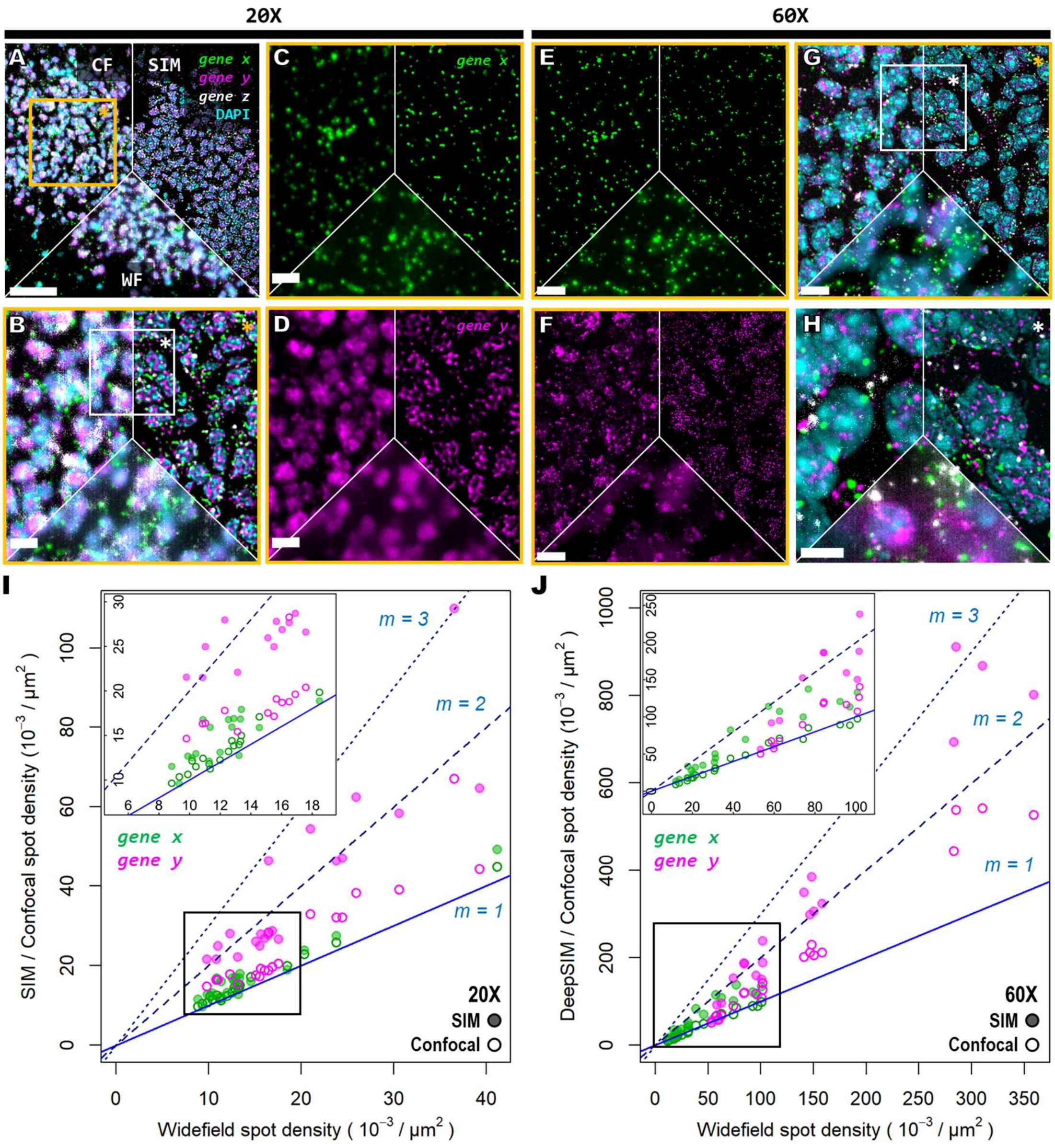

Figure S1. Structured illumination enhances spot contrast and resolution and allows resolving individual transcripts across various spot densities, magnification levels and imaging modalities. (**A**) Three different groups of 50 genes are visualized in each channel (Cy3, Cy5 and Cy7) referred to as *gene x*, *gene y* and *gene z* for simplicity. A single FOV of a mouse brain coronal section stained with DAPI is shown, imaged with a 20X objective. For panels (**A**) through (**H**), a comparison of the same FOV imaged in widefield (lower sector), spinning disk confocal (upper-right) and SIM (upper-right) modalities is shown. (**B**) Zoomed image of the yellow box in (**A**). Panels (**C**) through (**F**) correspond to the same zoomed FOV as in (**B**), displaying only the channels for either *gene x* (upper row) or *gene y* (lower row) alone, both in 20X (**C**,**D**) and 60X (**E**,**F**) magnification. (**G**) FOV corresponding to the region inside the yellow box in (**A**), but imaged with a 60X objective. (**H**) Zoomed image of the white box in (**G**) and (**B**). In all panels, color-wise asterisks indicate micrographs corresponding to the same physical region of the sample. Plots show the ratio of spot detection density when comparing imaging of *gene x* (green dots) and *gene y* (magenta dots) in widefield against spinning disk confocal (hollow dots) and SIM (colored dots) modes, both for the 20X (**I**) and 60X (**J**) objectives. Blue and dotted blue lines correspond to an increasing spot density ratio from 1 to 3. The inner plot shows a zoomed version of the regions in the black boxes. Scale bars: (**A**) = 50 µm; (**B**,**C**,**D**,**E**,**F**) = 10 µm; (**H**) = 5 µm.

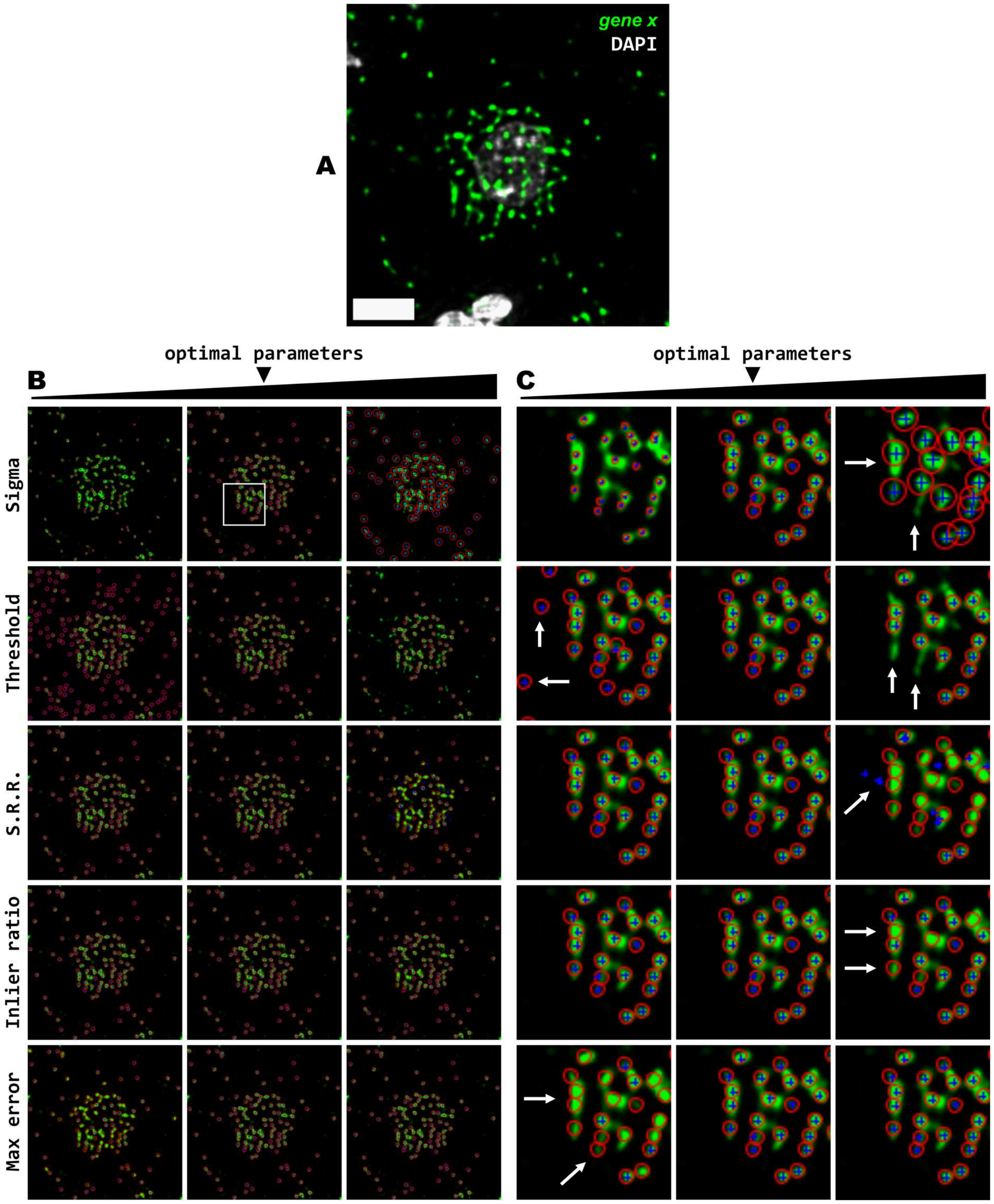

Figure S2. Effect of RS-FISH parameters tuning over the efficiency and accuracy of spot localization. (**A**) Representative example of a cell with fluorescently labelled amplified transcripts. (**B**) The effect of the tuning of each localization parameter is shown. Central column corresponds to the ideal case where the parameters allow the correct localization of most spots in the image while avoiding false positives and background signal. Left and right columns correspond to the scenario where each parameter was too low or too high, respectively. (**C**) Zoomed view of spot localizations, representing the region depicted by the white box in (**B**). Examples of incorrectly localized spots due to suboptimal parameter tuning are indicated with white arrows. Scale bar: 10 μm.

Adcyap1	*Esr1*	*Pvalb*	*Npy2r*	*Glul*	*Rorb*	*Tafa1*
Adipoq	*Nrxn1*	*Rbfox3*	*Ntrk2*	*Grin1*	*Rprm*	*Tpbg*
Agt	*Nxph1*	*Slc17a6*	*Oxt*	*Lypd1*	*Satb2*	*Vip*
Cartpt	*Pnoc*	*Slc17a7*	*P2rx3*	*Mup5*	*Syt6*	*Vipr2*
Cbln1	*Nxph4*	*Slc6a1*	*Pcp4*	*Neurod6*	*Adarb2*	*Adarb2*
Cbln4	*Ptprn2*	*Slc6a3*	*Pdyn*	*Oprk1*	*Arhgap36*	*Arhgap36*
Dbi	*Gls2*	*Slc6a4*	*Plp1*	*Pcp4*	*Calb1*	*Calb1*
Edn3	*Pcsk2*	*Slc6a5*	*Ptprc*	*Penk*	*Calb2*	*Calb2*
Gal	*Nmb*	*Sncg*	*Pvalb*	*Plcxd2*	*Cck*	*Cck*
Grp	*Apln*	*Spp1*	*Rbfox3*	*Ppp1r1b*	*Chodl*	*Chodl*
Igf1	*Ache*	*Sst*	*Slc17a6*	*Reln*	*Chrna2*	*Chrna2*
Igf2	*Acta2*	*Syn1*	*Slc17a7*	*Rorb*	*Cnmd*	*Cnmd*
Nos1	*Aldoc*	*Th*	*Slc6a1*	*Rprm*	*Crh*	*Crh*
Nucb2	*Bdnf*	*Tph1*	*Slc6a3*	*Satb2*	*Crhr2*	*Crhr2*
Oprd1	*Calca*	*Trh*	*Slc6a4*	*Syt6*	*Crispld2*	*Crispld2*
Oprl1	*Chat*	*Trpv1*	*Slc6a5*	*Tbr1*	*Egfr*	*Egfr*
Oprm1	*Dcn*	*Tubb3*	*Sncg*	*Trhr*	*Gabra1*	*Gabra1*
Scg2	*Fev*	*Vip*	*Spp1*	*Wfs1*	*Gabra2*	*Gabra2*
Scg3	*Fos*	*Ache*	*Sst*	*Adamts2*	*Gad1*	*Gad1*
Tacr1	*Gad1*	*Acta2*	*Syn1*	*Adora2a*	*Gad2*	*Gad2*
Ubl5	*Gad2*	*Aldoc*	*Th*	*Bcl11b*	*Hpse*	*Hpse*
Vgf	*Gfap*	*Bdnf*	*Tph1*	*Cbln2*	*Igfbp4*	*Igfbp4*
Slc32a1	*Gja1*	*Calca*	*Trh*	*Chrna6*	*Krt73*	*Krt73*
Grm1	*Itgam*	*Chat*	*Trpv1*	*Cpa6*	*Lamp5*	*Lamp5*
Grm3	*Kcnj8*	*Dcn*	*Tubb3*	*Cux1*	*Lhx6*	*Lhx6*
Grm4	*Laptm5*	*Fev*	*Vip*	*Cux2*	*Lmo1*	*Lmo1*
Grm7	*Map2*	*Fos*	*Adamts2*	*Deptor*	*Npy*	*Npy*
Htr2c	*Mbp*	*Gad1*	*Adora2a*	*Drd1*	*Nrtn*	*Nrtn*
Htr1a	*Ndnf*	*Gad2*	*Bcl11b*	*Fezf2*	*Nts*	*Nts*
Htr1f	*Nefh*	*Gfap*	*Camk2a*	*Foxp2*	*Plch2*	*Plch2*
Htr2a	*Neurod1*	*Gja1*	*Cbln2*	*Grin1*	*Prox1*	*Prox1*
Glra1	*Npy*	*Itgam*	*Chrna6*	*Lypd1*	*Pthlh*	*Pthlh*
Glra2	*Npy2r*	*Kcnj8*	*Cpa6*	*Mup5*	*Pvalb*	*Pvalb*
Glra3	*Ntrk2*	*Laptm5*	*Cux1*	*Neurod6*	*Rspo4*	*Rspo4*
Glra4	*Oxt*	*Map2*	*Cux2*	*Oprk1*	*Sema3e*	*Sema3e*
Glrb	*P2rx3*	*Mbp*	*Deptor*	*Pcp4*	*Slc17a8*	
Drd2	*Pcp4*	*Ndnf*	*Drd1*	*Penk*	*Sncg*	
Drd4	*Pdyn*	*Nefh*	*Fezf2*	*Plcxd2*	*Sst*	
Pnmt	*Plp1*	*Neurod1*	*Foxp2*	*Ppp1r1b*	*Tac1*	
Chrnb2	*Ptprc*	*Npy*	*Gls*	*Reln*	*Tac2*	

Table S1. Gene panel used for the ISS experiments.

	20X	25X	60X
WF	1.82 ± 0.131	1.26 ± 0.215	0.64 ± 0.312
DEC	1.34 ± 0.098	0.85 ± 0.076	NA
CF	1.71 ± 0.127	NA	0.58 ± 0.294
SIM	1.13 ± 0.112	0.68 ± 0.063	0.28 ± 0.206

Table S2. Measurement of spot FWHM for each objective magnification and imaging modality used. For each condition, at least 20 spots were manually measured using ImageJ. Reported values are mean ± std (µm). WF, Widefield; DEC, Deconvolution; SIM, Structured Illumination Microscopy; CF, Confocal.
